# Electrospinning/Electrospray of Ferrocene Containing Copolymers to Fabricate ROS-Responsive Particles and Fibers

**DOI:** 10.3390/polym12112520

**Published:** 2020-10-29

**Authors:** Hoik Lee, Jiseob Woo, Dongwan Son, Myungwoong Kim, Won Il Choi, Daekyung Sung

**Affiliations:** 1 Korea Institute of Industrial Technology, 143 Hanggaulro, Sangnok-gu, Ansan-si, Gyeonggi-do 15588, Korea; hoik@kitech.re.kr; 2 Center for Convergence Bioceramic Materials, Korea Institute of Ceramic Engineering and Technology, 202 Osongsaengmyeong 1-ro, Osong-eup, Heungdeok-gu, Cheongju, Chungbuk 28160, Korea; woooopaka@naver.com; 3 School of Chemical & Biomolecular Engineering, Yonsei University, 50 Yonsei-ro, Seodaemun-gu, Seoul 03722, Korea; 4 Department of Chemistry and Chemical Engineering, Inha University, Incheon 22212, Korea; sonob99707@gmail.com

**Keywords:** electrospinning/electrospray, ferrocene, amphiphilic polymer, nanofiber/nanoparticle, ROS responsiveness

## Abstract

We demonstrate an electrospray/electrospinning process to fabricate stimuli-responsive nanofibers or particles that can be utilized as stimuli-responsive drug-loaded materials. A series of random copolymers consisting of hydrophobic ferrocene monomers and hydrophilic carboxyl groups, namely poly(ferrocenylmethyl methacrylate-*r*-methacrylic acid) [poly(FMMA-*r*-MA)] with varied composition, was synthesized with free radical copolymerization. The morphologies of the resulting objects created by electrospray/electrospinning of the poly(FMMA-*r*-MA) solutions were effectively varied from particulate to fibrous structures by control of the composition, suggesting that the morphology of electrosprayed/electrospun copolymer objects was governed by its composition and hence, interaction with the solvent, highlighting the significance of the balance of hydrophilicity/hydrophobicity of the copolymer chain to the assembled structure. Resulting particles and nanofibers exhibited largely preserved responsiveness to reactive oxygen species (ROS) during the deposition process, opening up the potential to fabricate ROS-sensitive material with various desirable structures toward different applications.

## 1. Introduction

Research on local and temporary drug delivery systems (DDS) has gained considerable momentum in the development of new drug treatments [1]. As often observed in living organisms, many important functions are controlled by pulsed or temporarily released biologically active substances, in response to “demand” at specific times and sites [2,3]. In addition, many biologically active substances are being developed into more effective and complex treatment methods [1]. These treatment methods are usually rapidly metabolized in the body and may cause undesirable cytotoxic side effects when in large doses [4,5]. Thus, the novelty of a formulation that directly delivers drugs to diseased cells is identified as an essential factor for realizing the accuracy and specificity of treatment.

Remarkably, stimuli-responsive materials have newly programmable delivery systems where the release of the loaded drugs can be freely controlled by numerous intra- and extracellular biological stimuli (e.g., enzyme [6], pH [7], and redox potential [8]) in addition to external activations (e.g., temperature [9], light [10], and ultrasound [11]). Among them, materials responsive to a chemical stimulus such as reactive oxygen species (ROS) are ideal carriers because they could result in timely drug release patterns in specific physiological environments [12]. Ferrocene is a hydrophobic, organic compound used widely in the production of polymeric nanoparticles, and it allows for reversible self-assembly and controlled drug release [13]. In particular, with high levels of ROS, the hydrophobic neutral state of the ferrocene molecule undergoes oxidation and is rapidly converted to a hydrophilic ferrocenium cation, thus triggering drug release through the hydrophobic-to-hydrophilic transition [14]. Furthermore, in vivo safety of ferrocene has been demonstrated by clinical trials [15]. Thus, ferrocene-containing materials could be potential carriers for effective ROS-responsive drug delivery.

Until now, various research has been limited to polymersomes, hydrogels, and micelles [16]. Due to the importance of the fibrous and porous nanoscopic topography of the extracellular matrix (ECM) in facilitating basic cellular processes, new vehicles with biomimetic nanofiber properties are being considered [17]. Attractive drug transportation carriers are being progressed with a nanofibrous structure that enables maximum loading efficiency and low loading loss through uniform dispersion of drugs into the polymer matrix [1]. This type of nanofiber in sub-micron size can be fabricated with an electrospinning technique, which is regarded as one of most effective ways to produce incessant nanofibers from almost any supramolecules, composites, or polymers, which get tangled into a shape similar to polymers. The chance of using an electrospun nanofiber matrix as a concept to control the release of a wide variety of drugs, including anticancer drugs and antibiotics, has been explored [18].

**Figure 1:**
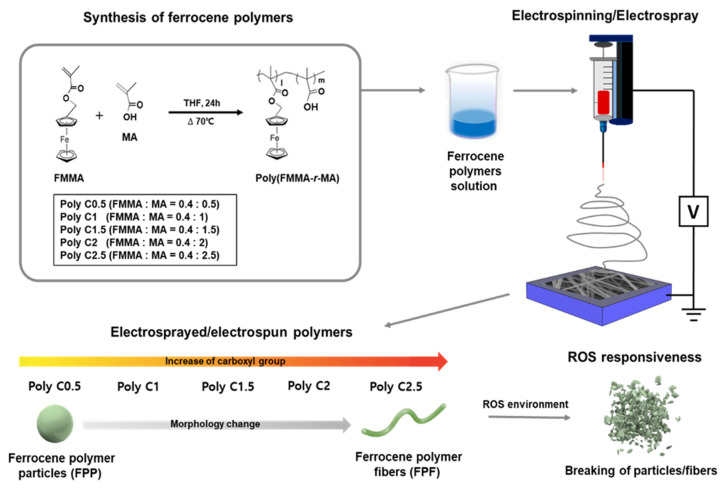
Schematics showing the synthesis of poly(FMMA*-r-*MA)) random copolymers and its use for fabricating a ferrocene polymer fiber (FPF) and a ferrocene polymer particle (FPP) with the electrospinning process.

The electrospinning technique utilizes interactions between an electrically charged liquid’s surface and a substrate. When a sufficiently high electric field is applied to a polymer droplet, an electrostatic repulsion force is generated on the liquid surface, counteracting its surface tension. As a consequence, the droplets ejected from the tip are stretched out to form nanofibers called electrospun nanofibers. On the other hand, when a stable liquid droplet on the tip is disintegrated into a number of droplets above the critical voltage, it is referred to as electrospray [19]. In the electrospray process, micro- or nanoscale particles are formed through instant and complete evaporation of the solvent during the flight of liquid droplets to the collector. It is well-known that the determination of electrospinning and electrospray is highly dependent on viscoelasticity and surface tension of the solution, which are controlled by its concentration [20]. The electrospray process leads to the formation of micro/nanoparticles, while the electrospinning process fabricates a fibrous structure. The processing parameters of the electrospinning/spray techniques have been investigated in various literature, which reports that solution concentration, applied voltage, solution feeding rate, tip-to-collector distance, and solubility parameter have varying effects on fiber or particle structure [21,22,23,24]. In addition, the interaction between polymer and solvent molecules, i.e., the type of solvent, largely affect the final morphology of the electrosprayed/electrospun structure [25]. Eventually, by control of the various parameters discussed above, the resulting morphology should be effectively controlled. Herein, we combined the electrospray/electrospinning techniques with stimuli-sensitive materials to fabricate stimuli-responsive drug-loaded nanofibers or particles. We previously developed ROS-responsive ferrocene nanoparticles formed by self-assembly of random copolymers consisting of hydrophobic ferrocene monomers and hydrophilic carboxyl groups, namely poly(ferrocenylmethyl methacrylate-*r*-methacrylic acid) [poly(FMMA-*r*-MA)], with outstanding release performance in ROS environments [13]. In the attempt to obtain a more efficient and specific system for developing ROS-responsive materials, ROS-responsive ferrocene polymer fiber (FPF) and ferrocene polymer particle (FPP) were fabricated with the electrospinning process, as illustrated in Figure 1. Interestingly, the morphologies of the resulting electrosprayed/electrospun poly(FMMA-*r*-MA), ranging from particulate to fibrous structure, were governed by copolymer composition and its interaction with the solvent, highlighting the significance of the hydrophilicity/hydrophobicity balance of the copolymer chain to the assembled structure during the deposition process. The responsiveness to ROS was preserved with the employed process, further opening up the feasibility to fabricate ROS-sensitive material with the desired structure.

## 2. Materials and Methods

### 2.1. Materials

Ferrocenylmethyl methacrylate (FMMA, 95%), methacrylic acid (MA, 99%), tetrahydrofuran (THF, anhydrous, 99.9%) and an inhibitor removal column were purchased from Sigma-Aldrich (St. Louis, MO, USA). 2,2-azobisisobutyronitrile (AIBN, 99%) was obtained from Daejung Chemical (Seoul, Korea). Deionized (DI) water was purchased from HyClone (Logan, UT, USA). Hydrogen peroxide aqueous solution (H_2_O_2_, 30%) was obtained from Junsei Chemical (Tokyo, Japan). All solvents were used as received without any further purification.

### 2.2. Characterization

The morphologies were examined using field emission scanning electron microscopy (FE-SEM, SU8010, Hitachi Co., Tokyo, Japan). All SEM specimens were coated with osmium using an ion coater (E-1045, Hitach, Tokyo, Japan) for 60 s prior to SEM imaging to enhance the conductivity of the samples. Elemental mapping analysis was accomplished using an energy-dispersive X-ray spectrometer (EDS, X-MAX 50, Oxford, UK) with the silicon drift detector (SDD) type (50 mm^2^ collection window). X-ray photoelectron spectroscopy (XPS) was conducted using a Thermo Scientific K-Alpha instrument (Waltham, MA, USA) with a monochromated Al Kα X-ray source at a takeoff angle to the substrate of 45°. Proton nuclear magnetic resonance (^1^H NMR, 400 MHz) spectra were recorded on a JEOL JNM-ECZ400S/L1 spectrometer (JEOL, Tokyo, Japan) using deuterated dimethyl sulfoxide (DMSO-d6) as the solvent at 25 °C. Molecular weight and dispersity (*Đ* = M_w_/M_n_) of the ferrocene-containing copolymer samples were obtained by size exclusion chromatography (SEC, 1200S/miniDAWN TREOS, Agilent Technologies, Santa Clara, CA, USA) at a flow rate of 1.0 mL/min at 35 °C with THF as the eluent. To verify the sensitivity to ROS of the electrosprayed particles and electrospun nanofibers, 0.1% hydrogen peroxide (H_2_O_2_) was added to the electrospun/electrosprayed samples as an oxidizing agent in aqueous solution (1 mg/mL) with gentle stirring for 4 h, followed by lyophilization to the obtained ROS-treated samples. Morphological and compositional changes upon oxidation were studied using SEM (TESCAN Mira3, Czech Republic) and XPS measurements.

### 2.3. Synthesis of Poly(FMMA-r-MA)

Ferrocene-containing copolymers were synthesized via radical polymerization, as previously reported. Prior to polymerization, MA was passed through the inhibitor removal column. As a typical procedure, FMMA (0.4 mmol) and MA (0.5 mmol) were dissolved in 2 mL of anhydrous THF, followed by AIBN addition (0.12 mmol). The mixture was then degassed with Ar gas bubbling for 5 min. The reaction mixture was stirred at 70 °C for 24 h for the polymerization reaction, followed by cooling down to below 25 °C and, finally, stored at 4 °C before use. Five ferrocene-containing copolymers with variations of the feed ratio of FMMA and MA monomers were synthesized: Poly C0.5 (molar ratio of FMMA to MA of 0.4:0.5), Poly C1 (0.4:1), Poly C1.5 (0.4:1.5), Poly C2 (0.4:2), and Poly C2.5 (0.4:2.5). The composition of the resulting copolymers was quantitatively analyzed with 1H NMR spectra by integrating the identified peaks as follows: δ = 4.8 (br, 2H, CH_2_ of FMMA), 4.1–4.4 (br, 9H of FMMA), 3.3–3.7 (br, 20H), 2.5–2.7 (br, 18H), 1.7–2.0 (br, 15H), and 0.8–1.1 (br, 17H) ppm.

### 2.4. Electrospinning of Ferrocene-Containing Polymers

All ferrocene-containing polymer solutions for electrospinning were prepared in THF with the concentration of 20 wt% to investigate the electrospinning behavior, depending on copolymer composition. An electrospinning apparatus (ESR200RD, NanoNC, Seoul, Korea) was equipped with a 30 kV high-voltage generator and drum-type collector (NNC-DC90H, NanoNC, Seoul, Korea). The prepared copolymer solution was injected into a plastic syringe containing a metallic needle (tip gauge: 21), and the injection rate was precisely controlled with a syringe pump. The process parameters, voltage, tip-to-collector distance, and flow rate were set to 20 kV, 10 cm, and 1 mL/h, respectively. All electrospinning experiments were performed at room temperature with relative humidity of approximately 40%.

## 3. Results and Discussion

### 3.1. Morphology Studies

The resulting morphology of an electrospun/electrosprayed sample is governed by various parameters, i.e., concentration of polymer solution, solvent quality, and various spinning process parameters. Particularly, solvent quality, i.e., the interaction between a polymer chain and solvent molecules, is a significant factor in determining the morphology. This effect on the final morphology of electrosprayed particles or electrospun nanofibers was effectively described with the compatibility of a polymer with different solvents. In electrospinning and electrospraying processes, the applied electrodynamic force overcomes the surface tension of the polymer solution, and as a result, the solution droplet is deformed to make Taylor cone-jet. If the applied electric force is sufficiently intense, a distorted hemispherical liquid drop is formed at the end of the top. Above the critical voltage, a liquid jet at the end of the capillary disintegrates into a number of small droplets that are sprayed to form particles or stretched out to form a nanofiber. Solvent quality, i.e., the interaction between polymer chains and solvent molecules, significantly affects the viscoelasticity of the solution and hence, the final morphology is also affected.

**Figure 2:**
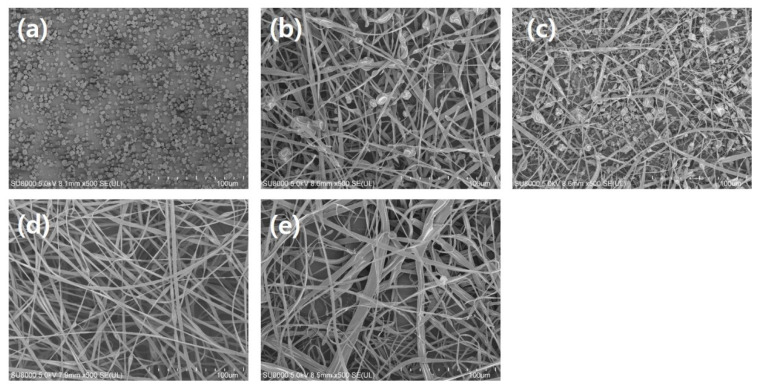
SEM images of electrospun/sprayed poly(FMMA-*r*-MA)s of (**a**) Poly C0.5, (**b**) Poly C1, (**c**) Poly C1.5, (**d**) Poly C2, and (**e**) Poly C2.5.

The random copolymers consisted of FMMA and MA units with varied compositions, and the FMMA and MA units were expected to be hydrophobic and hydrophilic, respectively. Therefore, the compositional variation also systematically controlled the interaction between the copolymer and the solvents. This effect is clearly observed in Figure 2, showing SEM images of the resulting morphologies of electrospun/sprayed poly(FMMA-*r*-MA)s with different compositions. Poly C0.5 units, having the richest amount of FMMA, exhibited a particulate structure during the process (Figure 2a). As the amount of MA units increased, the resulting structure, upon electrospinning, changed to a fibrous structure. Poly C1 with amount of MA double than that of Poly C0.5 exhibited a mixed morphology of beads and fibers (Figure 2b). Poly C1.5 showed a structure similar to Poly C1, with irregular and bead-containing fibers (Figure 2c). The solubility parameter of THF was 19.4 MPa^1/2^, while that of poly(MA) was 26.7 MPa^1/2^ [26,27]. Regarding the use of THF to produce poly(FMMA) and thermodynamic compatibility with other polymers [28,29], it was expected to be a compatible solvent with poly(FMMA). Hence, MA units in the copolymer chains tended to interact with each other to assemble into a specific structure. In this process, the relative amount of MA units in the copolymer chain played an important role: in the solution of Poly C0.5, the amounts of hydrophilic and hydrophobic components are similar, making the interaction between the copolymer chains balanced. A relatively large amount of FMMA led to effective interaction of a large portion of copolymer chains with the solvent molecules and, consequently, particulate morphology was favored. However, when the portion of MA units in the copolymer chain increased, the copolymers exhibiting unbalanced composition highly interacted with other copolymer chains in the system. Due to a large portion of MA units in the copolymer chain in Poly C2.5, the MA units in the copolymer chain likely interacted with the MA units in other copolymer chains rather than the solvent molecules, leading to the association of the copolymer chains to form a transient network [30,31], amplifying surface tension enough for the Poly C2.5 solution to be drawn to a fibrous structure (Figure 2e) [32]. As a consequence, the structure transformed from bead to nanofiber. Thus, the formation of an irregular bead-containing fiber structure in Poly C1 suggested that enough MA units existed in the system to withstand the drawing force applied by the electric field. Therefore, copolymers with larger amounts of the MA unit, i.e., Poly C1.5, Poly C2, and Poly C2.5, exhibited the fibrous structure (Figure 2c–e). In particular, Poly C2 showed bead-free nanofibers with excellent size uniformity, compared to Poly 1.5 and Poly 2.5. These results elucidated that the change of copolymer chain polarity and relative affinity of the solvent affect electrospray/spinning behavior in a concerted manner and as a consequence, the morphology was systematically changed.

**Figure 3:**
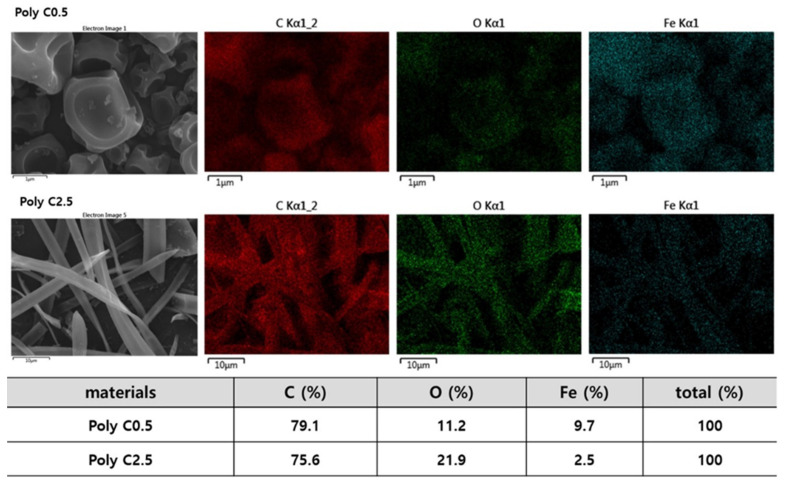
SEM images and corresponding elemental mapping results for C, O, and Fe for electrospun/sprayed Poly C0.5 and Poly C2.5 samples, and obtained atomic concentrations.

**Figure 4:**
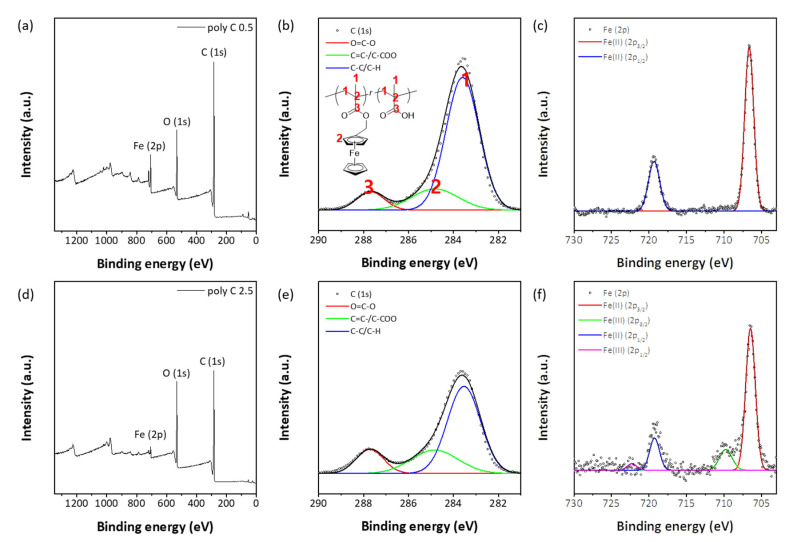
XPS spectra of electrospun/sprayed poly C0.5 and poly C2.5 samples, (**a**,**d**) survey spectra, (**b**,**e**) C_1s_ multiplex spectra, and (**c**,**f**) Fe_2p_ multiplex spectra, respectively.

Chemical compositions of the resulting particles and fibers were investigated further using EDS and XPS measurements. Figure 3 shows the EDS results of two representative samples of Poly C0.5 and Poly C2.5. Elemental mapping with SEM images clearly showed homogeneous distributions of three elements, C, O, and Fe, in the resulting particles and fibers. Quantitative EDS analysis results are also shown in Figure 3: the concentration of Fe in Poly C2.5 fiber was found to be higher than that in Poly C0.5, which was expected, as the Poly C2.5 copolymer chain has fewer FMMA units. Estimated atomic concentrations of Poly C0.5 and Poly C2.5 with the composition obtained from ^1^H NMR quantitative analyses (Table 1) were 78.4% (C), 17.7% (O), 3.9% (Fe), and 72.2% (C), 25.9% (O), 1.9% (Fe), respectively. Comparing the concentrations obtained from EDS analysis with the theoretical compositions, the concentration of Fe tended to be higher than its theoretical values. XPS spectra were also closely examined: the survey spectra shown in Figure 4a,d showed three prominent peaks near 285, 530, and 710 eV, which were assigned to C_1s_, O_1s_, and Fe_2p_, respectively. C_1s_ peaks were closely examined with the XPS spectra obtained with high resolution (Figure 4b,e). The atomic concentrations of Poly C0.5 and Poly C2.5 were 79.3% (C), 18.0% (O), 2.7% (Fe), and 73.2% (C), 25.7% (O), 1.1% (Fe), respectively, in good agreement with the values from the copolymer compositions. Deconvolution of the C_1s_ peaks revealed that the amount of carbon-forming C-C and C-H bonds in Poly C2.5 was higher than that in Poly C0.5, which was expected considering the composition of both copolymers. It was noted that a small amount of Fe(III) was observed in Fe_2p_ multiplex spectra (Figure 4c,f), suggesting possible oxidation of a small portion of ferrocenyl groups during the electrospinning process.

### 3.2. Responsiveness on Reactive Oxygen Species

**Figure 5:**
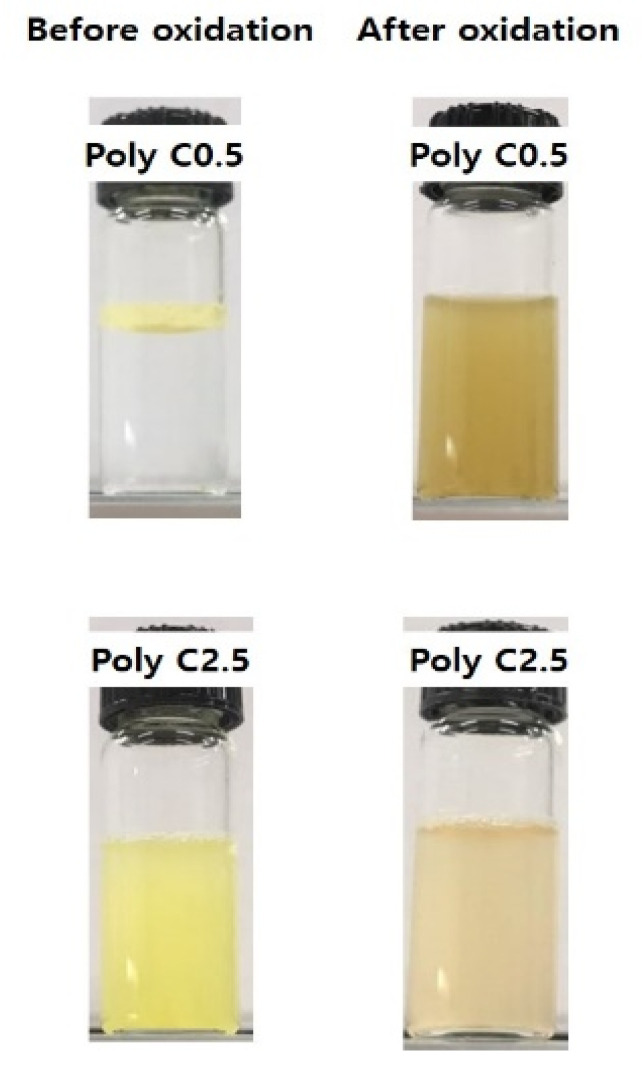
Photographs showing the dispersibility change of FPP (Poly C0.5) and FPF (Poly C2.5) upon exposure to H_2_O_2_.

The responsiveness to ROS of the fabricated poly(FMMA-*r*-MA) particles with Poly C0.5 (FPP) and fibers with Poly C2.5 (FPF) was examined by observing the changes in dispersibility and morphology with H_2_O_2_ as a ROS source. The FPP sample (Poly C0.5) did not interact with water, but FPF (Poly C2.5) was dispersed well due to its composition with a high concentration of MA units (Figure 5, left column). Upon addition of 0.1% H_2_O_2_ aqueous solution into each solution, both solutions turned to dark brown dispersion (Figure 5, right column). This change was known to be due to oxidation of the ferrocene group (Fe^2^⁺) into the positively charged ferrocenium group (Fe^3^⁺) by the reactive oxygen [33].

**Figure 6:**
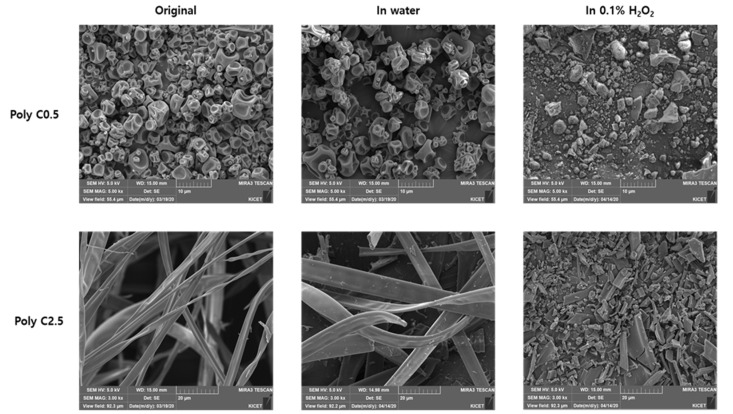
SEM images showing the morphological changes of FPP (Poly C0.5) and FPF (Poly C2.5) samples upon exposure to an ROS environment.

**Figure 7:**
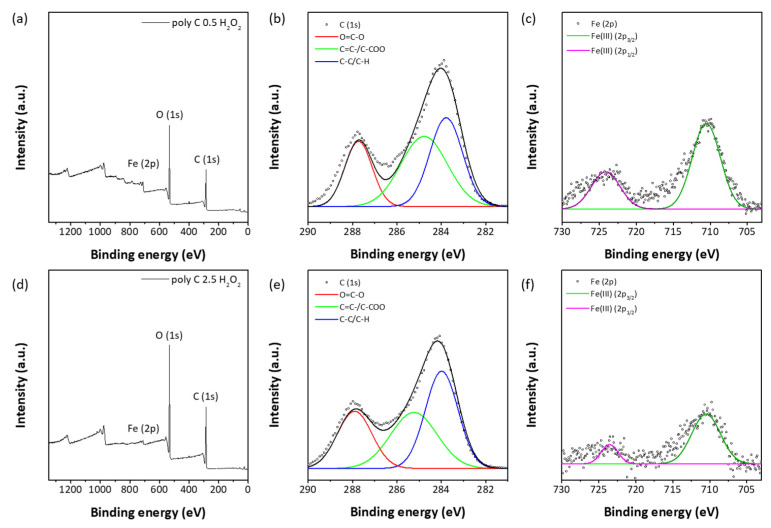
XPS spectra of FPP (poly C0.5) and FPF (poly C2.5) samples treated with H_2_O_2_ aqueous solution, (**a**,**d**) survey spectra, (**b**,**e**) C_1s_ multiplex spectra, and (**c**,**f**) Fe_2p_ multiplex spectra, respectively.

Morphological changes also were examined with SEM studies; Figure 6 shows pristine samples and samples treated with H_2_O and H_2_O_2_. Both samples did not show any changes upon treatment with H_2_O_2_. However, samples treated with the ROS solution showed significant morphological changes. In the case of FPP (Poly C0.5), the majority of particles was crushed to much smaller size. FPF (Poly C2.5) also showed destruction of the long thread-shaped fibers into small debris particles. Chemical compositions of FPP (Poly C0.5) and FPF (Poly C2.5) samples showed significant changes upon exposure to the ROS source. Both samples showed almost identical XPS C_1s_ multiplex spectra, as shown in Figure 7. By deconvolution of the spectra, the carbons with three different bonds were identified: C–C/C–H, C–COO, and O=C–O. The ratio of integrated intensities of the three carbons was close to 2:1:1, which matched well with the structure of MA units. Fe_2p_ multiplex spectra also changed largely upon ROS treatment, as shown in Figure 7c,f. The peaks assigned to Fe(II) almost disappeared, and only Fe(III) peaks were observed. These results strongly suggested that the ferrocenyl group was oxidized into the water-soluble ferrocenium group and hence, the ferrocenyl group in FMMA was removed to form a methacrylic acid unit. As a result, the copolymer chains began to effectively interact with water molecules, resulting in abrupt swelling and subsequent breaking of particles and fibers into smaller particles. ROS-responsiveness was not affected by the electrospray/electrospinning process to fabricate particles and fibers from poly(FMMA-*r*-MA) copolymers. Therefore, the particles and fibers have potential to be utilized as smart ROS-sensitive carriers for efficient drug delivery into target sites [13].

## 4. Conclusions

The morphology of ROS-responsive ferrocene-containing random copolymers, poly(FMMA-*r*-MA), was successfully controlled via electrospraying/electrospinning both particulate and fibrous structures by controlling the balance between hydrophobic and hydrophilic components. The compositional variation in copolymer structure was achieved by copolymerization of FMMA and MA monomers with controlled feed amounts. The compositional variation systematically and effectively changed the interaction between copolymer chains and solvent molecules. When the amount of FMMA units was large enough to effectively interact with solvent molecules, particulate morphology was observed upon electrospray of the polymer solution. However, when the portion of MA units becomes larger, the interaction between MA units becomes significant enough to form a transient network-like structure and provide enough surface tension for the copolymer solution to be drawn into a fibrous structure during the electrospray/eletrospinning process. In between the compositions, the hybrid structure of fiber and particles was observed. Furthermore, the properties of response to ROS were largely preserved in the electrospraying/electrospinning processes, as confirmed with EDS and XPS studies. Significant changes in dispersibility, morphology, and chemical composition of poly(FMMA-*r*-MA) particles and fibers upon treatment with H_2_O_2_ clearly confirmed effective oxidation of Fe(II) to Fe(III) with H_2_O_2_ in FMMA units. These results highlighted the potential of biocompatible poly(FMMA-*r*-MA) as a ROS-responsive material platform with desirable morphology and structure towards target biomedical applications where the effective release of a hydrophobic drug with the oxidation with ROS is necessary.

## Figures and Tables

**Table 1:** Characteristics of synthesized poly(FMMA-*r-*MA) samples.

Sample	*f*_FMMA_	*F*_FMMA_^a^	M_n_ (g/mol) ^b^	*Đ* ^b^
Poly C0.5	0.444	0.484	11,100	1.75
Poly C1	0.286	0.308	9800	2.15
Poly C1.5	0.211	0.236	11,100	2.03
Poly C2	0.167	0.184	9400	1.81
Poly C2.5	0.138	0.143	13,100	1.91

^a^ Determined with quantitative analyses of ^1^H NMR spectra; ^b^ Determined with SEC analyses with PS standard samples; *f*_FMMA_ is a composition of the FMMA monomer in the feed; *F*_FMMA_ is the actual composition of FMMA in the copolymer; M_n_ is average molecular weight; *Đ* is dispersity (M_w_/M_n_).
